# Emerging trends in invasive *Streptococcus dysgalactiae* subsp. *equisimilis* infections in Denmark, 2014 to 2024: a nationwide genomic and registry-based study

**DOI:** 10.2807/1560-7917.ES.2026.31.35.2500714

**Published:** 2026-09-03

**Authors:** Jana Grüttner, Aoife Ronayne, Raphael Niklaus Sieber, Thor Bech Johannesen, Zahraa Chayed, Esad Dzajic, Ulrich Stab Jensen, David Fuglsang-Damgaard, Marianna Konoy, Lars Lemming, Xiaohui Chen Nielsen, Emma Julia Petronella Nilsson Gram, Claus Østergaard, Michael Pedersen, Steen Hoffmann, Marc Stegger

**Affiliations:** 1 Department of Bacteria, Parasites and Fungi, Statens Serum Institut, Copenhagen, Denmark; 2 European Programme for Public Health Microbiology Training (EUPHEM), European Centre for Disease Prevention and Control (ECDC), Stockholm, Sweden; 3 Department of Sequencing and Bioinformatics, Statens Serum Institut, Copenhagen, Denmark; 4 Department of Clinical Microbiology, Odense University Hospital (OUH), Odense, Denmark; 5 Department of Clinical Microbiology, Esbjerg and Grindsted Hospital, Esbjerg, Denmark; 6 Department of Clinical Microbiology, Herlev-Gentofte University Hospital, Herlev, Denmark; 7 Department of Clinical Microbiology, Aalborg University Hospital, Aalborg, Denmark; 8 Rannsóknarstovan, Landssjúkrahúsið, Faroe Islands; 9 Department of Clinical Microbiology, Aarhus University Hospital, Aarhus, Denmark; 10 Department of Clinical Microbiology, Zealand University Hospital, Koege, Denmark; 11 Department of Clinical Microbiology, Rigshospitalet, Copenhagen, Denmark; 12 Department of Clinical Microbiology, Vejle Hospital, Vejle, Denmark; 13 Department of Clinical Microbiology, Copenhagen University Hospital - Amager and Hvidovre, Copenhagen, Denmark; 14 Antimicrobial Resistance and Infectious Diseases Laboratory, Harry Butler Institute, Murdoch University, Perth, Australia

**Keywords:** *Streptococcus dysgalactiae* subsp. *equisimilis*, Group C streptococci, Group G streptococci, AMR, surveillance, invasive infection, beta-haemolytic streptococci

## Abstract

**BACKGROUND:**

Increasing incidence rates of invasive *Streptococcus dysgalactiae* subspecies *equisimilis* (iSDSE) have been detected worldwide.

**AIM:**

We aimed to investigate iSDSE infection incidence rates in Denmark during 2014–2024, and characterise the genomic population structure of a subset of iSDSE isolates and their antimicrobial resistance (AMR).

**METHODS:**

Using national register data, we estimated overall and sex-/age-stratified iSDSE incidences during 2014–2024, by retrospectively identifying cases of invasive infections with group C and G streptococci or *S. dysgalactiae* (including specified as subspecies *equisimilis*). From the voluntary national beta-haemolytic streptococci laboratory surveillance system, whole-genome-sequenced isolates from August 2020−September 2022 were used to investigate the iSDSE genomic population structure. Susceptibility to penicillin, erythromycin and clindamycin was determined and AMR genes identified.

**RESULTS:**

During 2014–2024, iSDSE incidence rates increased significantly (linear trend analysis p < 0.001) with mean annual incidence ranging between 10.3 and 16.4 per 100,000, peaking in 2023. Incidence was higher in males, increasing with older age. Nearly 75% of the 1,223 iSDSE isolates belonged to four of 14 genetic clusters. Sequence types (STs) ST20 and ST17 were most prevalent, while *emm*-type *stG62647*, a variant associated internationally with higher virulence, dominated. All isolates were phenotypically susceptible to penicillin but approximately 10% were respectively erythromycin and clindamycin resistant. High erythromycin resistance prevalence (57%; 39/68), coinciding with gene *ermA*, occurred in one genetic cluster.

**CONCLUSION:**

The findings illustrate the need for national registry-based surveillance to detect epidemiological changes and potential outbreaks. Further, continuous genomic surveillance can monitor the occurrence and expansion of genetic clades and AMR genes.

**Key public health message**

**What did you want to address in this study and why?**

*Streptococcus dysgalactiae* subspecies *equisimilis* (SDSE) is an emerging pathogen that can cause severe invasive disease in humans. We aimed to assess if the incidence of invasive SDSE infections had increased in Denmark from 2014 to 2024 and to determine the most affected population groups. We also explored the genomic population structure of invasive SDSE and if antimicrobial resistance occurred, as this could lead to treatment failure.

**What have we learnt from this study?**

The incidence of invasive SDSE increased over 11 years, with infections mostly affecting older adults and males. Among the invasive SDSE isolates genomically characterised, four genetic clusters dominated. Overall, no resistance to penicillin was detected; however, around 10% of isolates were resistant to clindamycin and erythromycin antibiotics. In one genetic cluster (39 cases) erythromycin resistance was particularly high at 57%.

**What are the implications of your findings for public health?**

The increase in invasive SDSE infections warrants further investigations of its causes. We suggest enhancing national surveillance of these infections and registry-based data should be used to assess incidences. Although no resistance to penicillin, the first-line treatment for invasive SDSE infections, was detected, the resistance to second-line drugs clindamycin and erythromycin is concerning, especially for individuals with penicillin allergy.

## Introduction

*Streptococcus dysgalactiae* subspecies *equisimilis* (SDSE) is a Gram-positive bacterial pathogen that belongs to the group of beta-haemolytic streptococci. Traditionally, beta-haemolytic streptococci are serologically classified into Lancefield groups [1]. For SDSE, the main Lancefield group antigens are C or G, but this subspecies also expresses antigens of group A and L, albeit with much lower frequency [2,3]. Being a common skin and throat commensal, SDSE can nevertheless cause invasive and non-invasive infections in humans [4,5]. Disease manifestations can be diverse, ranging from milder skin and soft tissue infections and pharyngitis to severe and life-threatening necrotising soft tissue infections, toxic shock syndrome, endocarditis and bacteraemia [4-7].

Increasing incidence rates of invasive SDSE (iSDSE) infections have been observed since the early 2000s in multiple countries globally [8-11], including Denmark [12-14] and other Nordic countries, such as Norway and Finland [5,15-17]. However, population-based studies on incidence rates of iSDSE are limited and comparison between them and settings remains challenging, since laboratory reports often lack species-level information and only classify streptococcal isolates by Lancefield group antigen (e.g. group C streptococci (GCS) and group G streptococci (GGS)) [18,19]. Surveillance of iSDSE in Denmark is laboratory-based and relies on voluntary submission of isolates from the regional departments of clinical microbiology (DCM) to the national public health institute, Statens Serum Institut (SSI). Increasing incidence rates based on the voluntary submitted isolates have been reported from 2005 to 2023 for both GCS and GGS in Denmark [13,14].

Despite a penicillin-resistant clone having been isolated from blood cultures of three epidemiologically linked patients in Denmark between 2010 and 2012 [20], SDSE is typically susceptible to penicillin, which is the first-choice treatment option for infections with this pathogen. In contrast, resistance of SDSE to other antibiotic classes including tetracyclines, macrolides, and lincomycins is commonly reported globally [10,14,18,21-24].

Aside from Lancefield serogrouping, SDSE can be characterised by molecular typing schemes, including multilocus sequence typing (MLST) [25,26], based on seven genes, and *emm*-typing [27-29], based on the hypervariable region of the *emm* gene, a major *Streptococcus pyogenes* virulence factor gene. Both sequence types (ST) and *emm*-typing are limited in representing genetic diversity and genome sequencing has been proven to greatly facilitate phylogenetic resolution of SDSE strains [10,30,31]. Our current knowledge of the genetic diversity of iSDSE isolates in Denmark is limited due to the lack of whole-genome sequencing (WGS) studies. As part of the iSDSE laboratory-surveillance in Denmark, antimicrobial susceptibility testing and WGS to investigate the molecular epidemiology of iSDSE has been performed on all isolates received by SSI from 17 August 2020, however, routine sequencing of all iSDSE isolates was discontinued after 18 September 2022.

To assess the development of iSDSE infections in humans and to inform infection prevention strategies going forward, this study aimed to use data from the Danish microbiology database (MiBa) [32] to estimate registry-based incidence rates of iSDSE in the country over an 11-year period ending in 2024. A further objective was to explore the genetic structure of the Danish iSDSE population including antimicrobial resistance (AMR) to understand the molecular epidemiology and occurrence of resistances that could lead to treatment failure.

## Methods

### Registry-based surveillance

#### Data collection

Data were collected retrospectively from MiBa, a real-time repository of all microbiological test results reported mandatorily by all DCMs across Denmark. The dataset covered the period from 1 January 2014 to 31 December 2024 and was extracted on 4 February 2025. The MiBa extraction terms were group C streptococci, group G streptococci, *S. dysgalactiae*, and *S. dysgalactiae* ssp. *equisimilis*.

#### Invasive case and infection episode definitions

We defined an invasive case as a person identified by their Central Person Registry Number (CPR, which is the Danish unique personal identification number), with an invasive sample, i.e. a positive culture from a normally sterile site (combination of sample location and sample type), including blood, cerebrospinal fluid, joint or bone samples, deep, organ, or muscle tissues, normally sterile implant material and abscess samples. Our case definition is solely based on information obtained from MiBa and is lacking clinical criteria. An infection episode was defined as an invasive sample taken at least 30 days after the last positive invasive sample from the same individual. All invasive samples, irrespective of type, were weighted equally when defining infection episodes and after calculating invasive case numbers. Bacteraemia cases were defined as any positive blood sample with the same infection episode definition.

#### Incidence rates and related statistical analyses

We calculated incidence rates using the mean population of Denmark for the second quarter of the year as the denominator. The population data were retrieved from the Statistics Denmark (https://www.dst.dk/en/Statistik) database, which was accessed on 12 February 2025. Comparisons between female and male incidence rates per age category were done by pairwise proportion testing with Holm correction [33]. We assessed a trend over time for the annual mean incidence rates using linear regression. All statistical analyses were carried out in RStudio (v2023.06.2 + 561) using R v4.3.1 [34]. p values below 5% were considered statistically significant.

### Clinical isolates from the national laboratory-based surveillance

#### Notification of iSDSE and voluntary submission of isolates to the surveillance system

Since 27 October 2023 iSDSE is a notifiable disease in Denmark according to the executive order on notification of infectious diseases (BEK nr 1260) and as such laboratory notification to SSI is mandatory. However, the submission of isolates from the DCMs to SSI is voluntary, except for iSDSE that has caused meningitis. The change in legislation does not affect how the SDSE data are recorded in MiBa. The current national iSDSE surveillance in Denmark is laboratory based and still relies on the voluntarily submitted isolates. The isolates are submitted with additional information, which includes specimen type, sampling date, CPR number, patient age, and sex (collected as male or female). Age comparisons between female and males were done by Wilcoxon rank sum test. All isolates that SSI received between 17 August 2020 and 18 September 2022 were genome sequenced.

#### Invasive case, infection episode and polyclonal infection definitions

The same invasive case definition was applied as for the registry-based surveillance cohort, i.e. a patient with an invasive sample. The same infection episode definition was applied to records in the registry-based cohort and the SSI cohort (30 days between positive samples), however, for a given infection episode, consecutive isolates yielding genetically distinct strains were classified as such, i.e. comprising a polyclonal infection. Genetic distance was determined using core-genome single nucleotide polymorphism (SNP) analysis (described below) and strains with high pairwise SNP distance were retained (> 100 SNPs).

#### Susceptibility testing of isolates

As part of the routine surveillance, all submitted isolates were subjected to susceptibility testing, and Lancefield serogroup classification. Antibiotic susceptibility was determined by disc diffusion method for penicillin (1 µg), erythromycin (15 µg), and clindamycin (2 µg) using Neo-Sensitabs (Rosco Diagnostica, Denmark) on Mueller−Hinton (MH) agar supplemented with 5% defibrinated horse blood and 20 mg/L β-Nicotinamide adenine dinucleotide (β-NAD) (i.e. MH medium supplemented for fastidious organisms; MH-F) (SSI Diagnostica, Denmark) incubated at 35°C with 5% CO_2_. Inducible clindamycin resistance was detected by performing double-disk diffusion testing (D-test) using clindamycin (2 µg) and erythromycin (15 µg) disks according to guidelines described in the clinical breakpoints for *Streptococcus* groups A, B, C and G in European Committee on Antimicrobial Susceptibility Testing (EUCAST) v14.0 (2024) (http://www.eucast.org/clinical_breakpoints/). Minimum inhibitory concentrations (MICs) for penicillin, erythromycin, and clindamycin were determined using the gradient strip method (ETEST; bioMérieux, France) for all non-susceptible isolates, and interpreted using clinical breakpoints for *Streptococcus* groups A, B, C and G in EUCAST v14.0 (2024). Lancefield group determination was performed by latex agglutination tests (Oxoid, United Kingdom (UK)).

### Genome sequencing and bioinformatic analysis

#### Library preparation, sequencing, species validation and genome assembly

DNA was purified using the DNA and Viral NA small volume kit on the MagNa Pure 96 system (Roche Diagnostics, Rotkreuz, Switzerland) and quantified using Quant-iT-dsDNA BR and HS assays (ThermoFisher Scientific, Waltham, United States (US)). Samples were prepared for WGS using half volume of the Illumina Nextera XT library preparation (Illumina, San Diego, US) on the Microlab STAR liquid handling workstation from Hamilton (Hamilton, Reno, US). The samples were sequenced on the Illumina NextSeq 550 platform (mid-out 300 cycles). We performed quality control (QC) of the data using an in-house QC pipeline (https://github.com/ssi-dk/bifrost), which included sequencing depth (minimum 50-fold coverage), species validation, and detection of contaminants (correct species identification > 85%). Genome assembly was performed using SKESA v2.2 [35], where assemblies with more than 400 contigs were excluded.

#### Assessment of isolate relatedness

We determined the relatedness of isolates using NASP v1.2.1 [36] to identify SNPs using the SDSE strain FDAARGOS_1017 (National Center for Biotechnology Information (NCBI) RefSeq assembly: GCF_016128095.1) as reference. Positions with < 90% unambiguous variant call and a depth of < 10-fold coverage in individual isolates were excluded. Regions of putative recombination were removed from the core-genome SNP alignment using Gubbins v3.3.1 [37]. The resulting alignment file was used to define genomic clusters using fastBAPS v1.0.8 [38] with the ‘baps’ prior optimisation option. Maximum likelihood (ML) phylogenetic trees were created using IQ-TREE v2.3.6 using the best model as estimated by the included model finder [39] (see illustrations with trees that follow) or a ML approximation was performed in FastTree v2.1.11 [40], as in the phylogenies displayed in Supplementary Figure S1. Genetic relatedness was visualised using ggtree (v3.8.2). Heatmap of pairwise SNP distances was created using heatmap.2 from the R package gplots (v3.2.0). Comparisons between age and between female and male per BAPS cluster was done by Kruskal−Wallis rank sum test and by proportion testing with Holm correction, respectively. Comparison of the study isolates with the SDSE isolates of human and animal origin from a Norwegian study of Porcellato *et al*.*,* 2021 (BioProject PRJEB43000) [41] were performed as described above, see Supplementary Data S1.

#### Determination of sequence and *emm* types

The k-mer alignment (KMA) v1.4.9 [42] tool was used to identify STs, with the pubMLST *S. dysgalactiae* (https://pubmlst.org/organisms/streptococcus-dysgalactiae) database accessed on 05 April 2024 [25]. *Emm*-types were identified using basic local alignment search tool (blast) v2.12.0 using the *emm* database (https://www.cdc.gov/strep-lab/php/group-a-strep/emm-typing.html) accessed on 05 April 2024.

#### Identification of antibiotic resistance genes and mobile genetic elements

Antibiotic resistance genes were detected by mapping the sequence reads against the ResFinder database v4.2.3 [43] using the KMA tool with a detection threshold of 90% sequence similarity and coverage. Mobile genetic elements were detected and analysed using ICEscreen v1.3.2 [44] and ICEberg3.0 [45] using genome draft assemblies generated by SPAdes v3.11.1 [46]. Illustration of integrative and conjugative element (ICE) was done using R package gggenes v0.5.1.

## Results

### Registry-based incidence rates of invasive SDSE infections

Because current surveillance of iSDSE in Denmark is based on voluntary submission of iSDSE isolates from the regional DCMs to SSI, MiBa data were used to get a better estimate of the incidence rates of iSDSE in the country. For this, culture-positive iSDSE tests records were extracted from the MiBa register.

**Figure 1:**
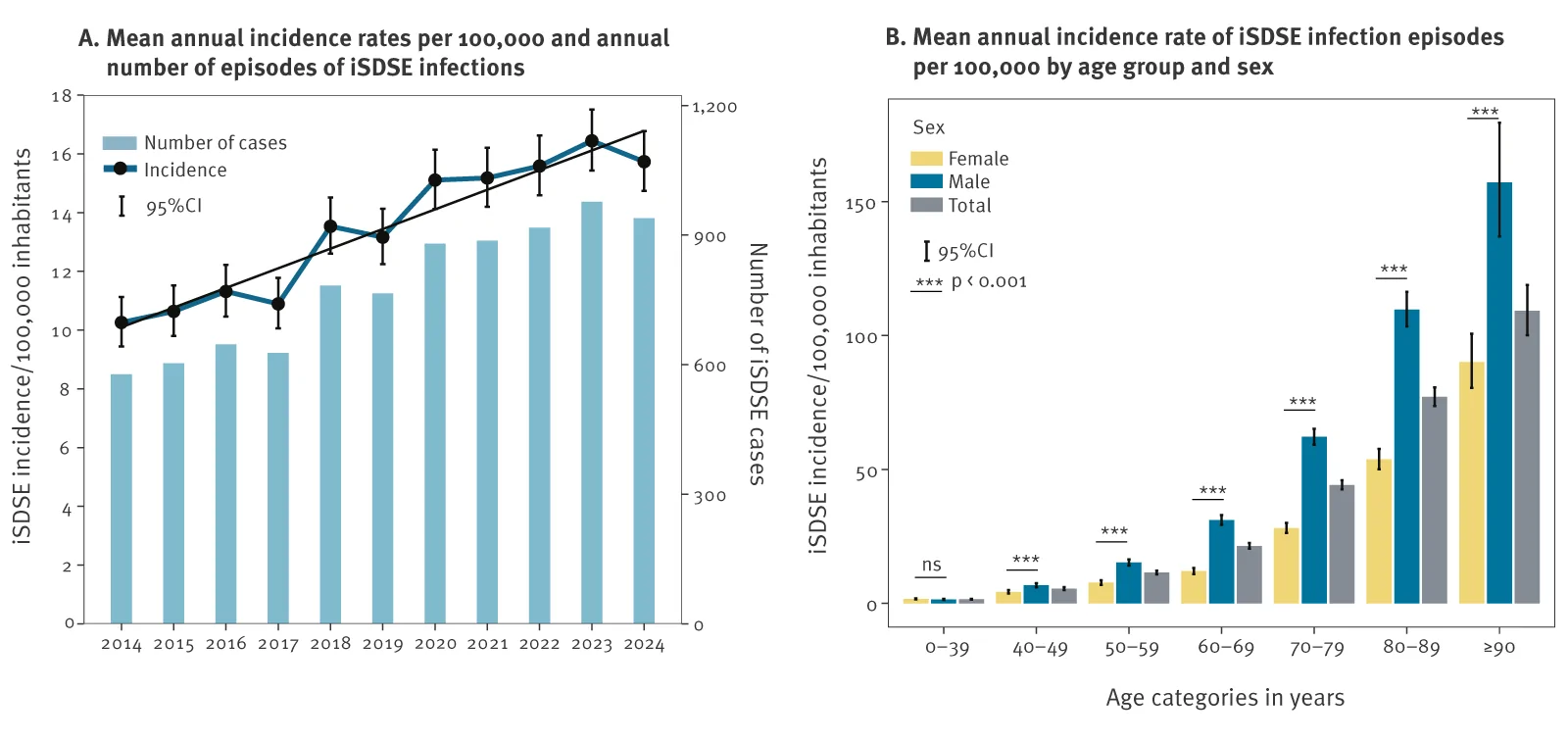
Registry-based (A) annual number of episodes and incidence rates of iSDSE overall and (B) by age and sex, Denmark, 2014−2024 (n = 8,603)

CI: confidence interval; iSDSE: invasive *Streptococcus dysgalactiae* subsp. *equisimilis*; ns: non-significant.

Between 2014 and 2024, we detected 8,603 iSDSE episodes among 7,949 individual patients, with 6.6% (n = 524) patients experiencing one or more recurrent infections. This is further detailed in Supplementary Table S1. The annual incidence rate increased significantly over the years (p < 0.001) from 10.3 per 100,000 inhabitants in 2014 to 15.7 per 100,000 inhabitants in 2024, with gradual year on year increases punctuated by small reductions in incidence in years 2016–2017, 2018–2019 and 2023–2024 (Figure 1A). The highest annual incidence rate was detected in 2023 with 16.4 per 100,000 inhabitants.

**A**. A linear regression trend line depicted in black shows the evolution of the mean annual incidence rates of infection episodes per 100,000, which are shown with 95% CI. The annual number of episodes of iSDSE infections are displayed by chart bars.

**B.** Mean annual incidence rate of iSDSE infection episodes per 100,000 per age category and sex are represented with 95% CIs. Statistical significance was determined using pairwise proportion testing with Holm-type corrections for multiple testing. Differences in iSDSE infection incidences between males and females in each age group are considered significant when p is < 0.05.

The median age of the cases was 73 years (interquartile range (IQR): 61–81) and the incidence rates increased in each successive age category overall and in both males and females (Figure 1B). Males accounted for 63% (n = 5,417) of cases. The incidence rate was higher in males than females during the entire study period and was significantly higher in all age categories except for cases in age category 0–39 years, as shown in Figure 1B and Supplementary Figure S2B.

To facilitate comparisons with previous international studies, we also calculated incidence rates of bacteraemia episodes (bSDSE) specifically. The annual incidence rate of bSDSE increased as well over the years, ranging between 6.9 and 12.4 per 100,000 inhabitants in 2014 and 2023, respectively (Supplementary Figure S2C).

### Molecular epidemiology of invasive SDSE

Between 17 August 2020 and 18 September 2022, SSI received 1,291 iSDSE isolates as part of the national laboratory surveillance, all of which were genome sequenced and characterised in terms of the Lancefield group and antimicrobial susceptibility. Three Lancefield group A isolates were also identified in the study period as iSDSE in the Danish genomic *S. pyogenes* surveillance programme and were therefore included in the study cohort, as illustrated in Supplementary Figure S3A.

Sequenced isolates that did not pass the quality control (n = 11) or were not identified as SDSE (n = 29; *Streptococcus anginosus* (n = 3), *Streptococcus equi* (n = 11) and *Streptococcus pyogenes* (n = 15)) were excluded.

Further, 29 isolates were excluded after applying an infection episode of a 30-day period after the last identified isolate from the same individual, except if the isolates were genetically different (> 100 SNPs). We identified two individuals who were infected with two genetically distinct iSDSE strains in the same infection episode (SNP differences of > 2,000) (Supplementary Figure S3B). The final study population comprised 1,225 high-quality iSDSE genomes.

The iSDSE isolate study cohort was derived from 1,172 distinct individuals of whom 43 had recurrent infections in the study period, as described in Supplementary Table S2.

The iSDSE isolates phenotypically classified as 61% Lancefield groups G (n = 742), 39% C (n = 480) and 0.2% A (n = 3). Also, 63% (n = 768) of the isolates were from male individuals and 37% (n = 457) from female individuals. The median age was 77 years (IQR: 68–83) and there was no significant difference in age between males and females, as indicated in Supplementary Table S3.

A comparison of the genomic study cohort, consisting of isolates voluntarily submitted to SSI (1,218 infection episodes, excluding Greenland, the Faroe Islands, and polyclonal infections) with the iSDSE cases identified via MiBa (1,892 infection episodes in the same study period 17 August 2020 and 18 September 2022), indicated that the number of cases with available genome sequencing data represented ca 64% of the total registry-detected cases during the same time period.

### Molecular typing and phylogeny

**Figure 2:**
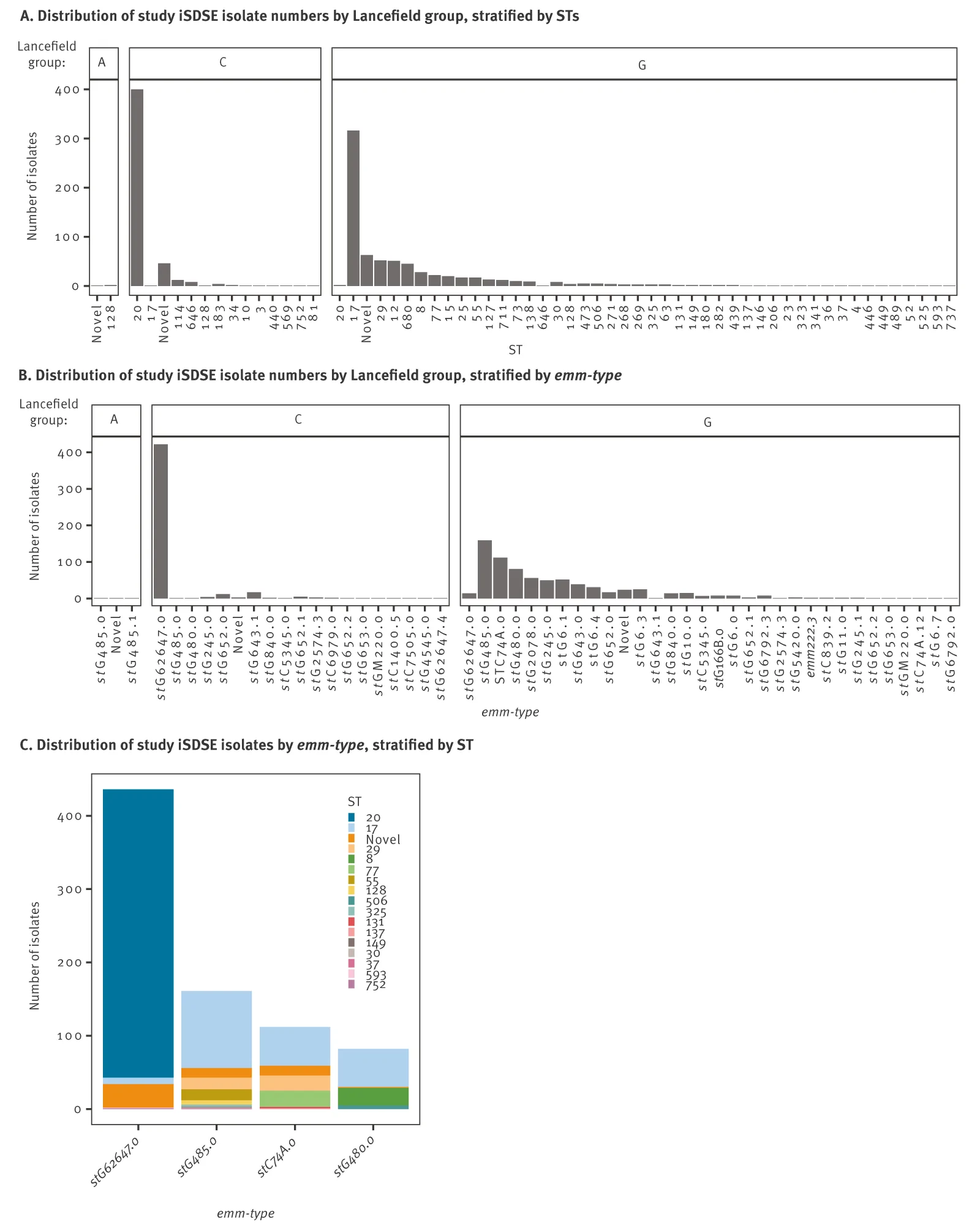
Genotype distribution of the iSDSE study population, by (A) Lancefield group and ST (B) Lancefield group and *emm*-type and (C) major *emm*-type and ST, Denmark (n = 1,220), Greenland (n = 1) and Faroe Islands (n = 4), 17 August 2020–18 September 2022

*In silico* genotyping of the isolates yielded a total of 54 STs and 37 *emm*-types. The predominant STs identified were ST20 (n = 402, 33%) and ST17 (n = 317, 26%), collectively accounting for over half of the sequenced population. A total of 52 other distinct STs were identified in genomic analyses, each comprising less than 5% of the total genomes identified among genotyped isolates, indicating a greater genomic diversity in the remaining population. The majority of ST20 (400/402; 99.5%) belonged to Lancefield group C, while the majority of ST17 belonged to Lancefield group G (316/317, 99.7%) (Figure 2A). The most prevalent *emm*-types were *stG62647.0* (n = 436, 35.8%), *stG485.0* (n = 161, 13.2%), *stC74A.0* (n = 112, 9.2%), and *stG480.0* (n = 82, 6.7%) (Figure 2B). Of note, 90.1% (393/436) of the dominant *emm*-type *stG62647.0* belonged to ST20. The other prevalent *emm*-types, *stG485.0*, *stC74A.0* and *stG480.0*, included a greater variety of STs but all had ST17 as the largest contributor (65.2% (105/161), 47.3% (53/112), and 63.4% (52/82), respectively), see Figure 2C.

iSDSE: invasive *Streptococcus dysgalactiae* subsp. *equisimilis*; ST: sequence type.

**A−B.** There were three isolates belonging to Lancefield group A, 480 to Lancefield group C and 742 to Lancefield group G.

The isolates for Denmark arise from 1,218 infection episodes, with two of these episodes involving two genetically distinct iSDSE strains (with single nucleotide polymorphism differences of > 2,000), thus the total number of Danish isolates is 1,220.

**Figure 3:**
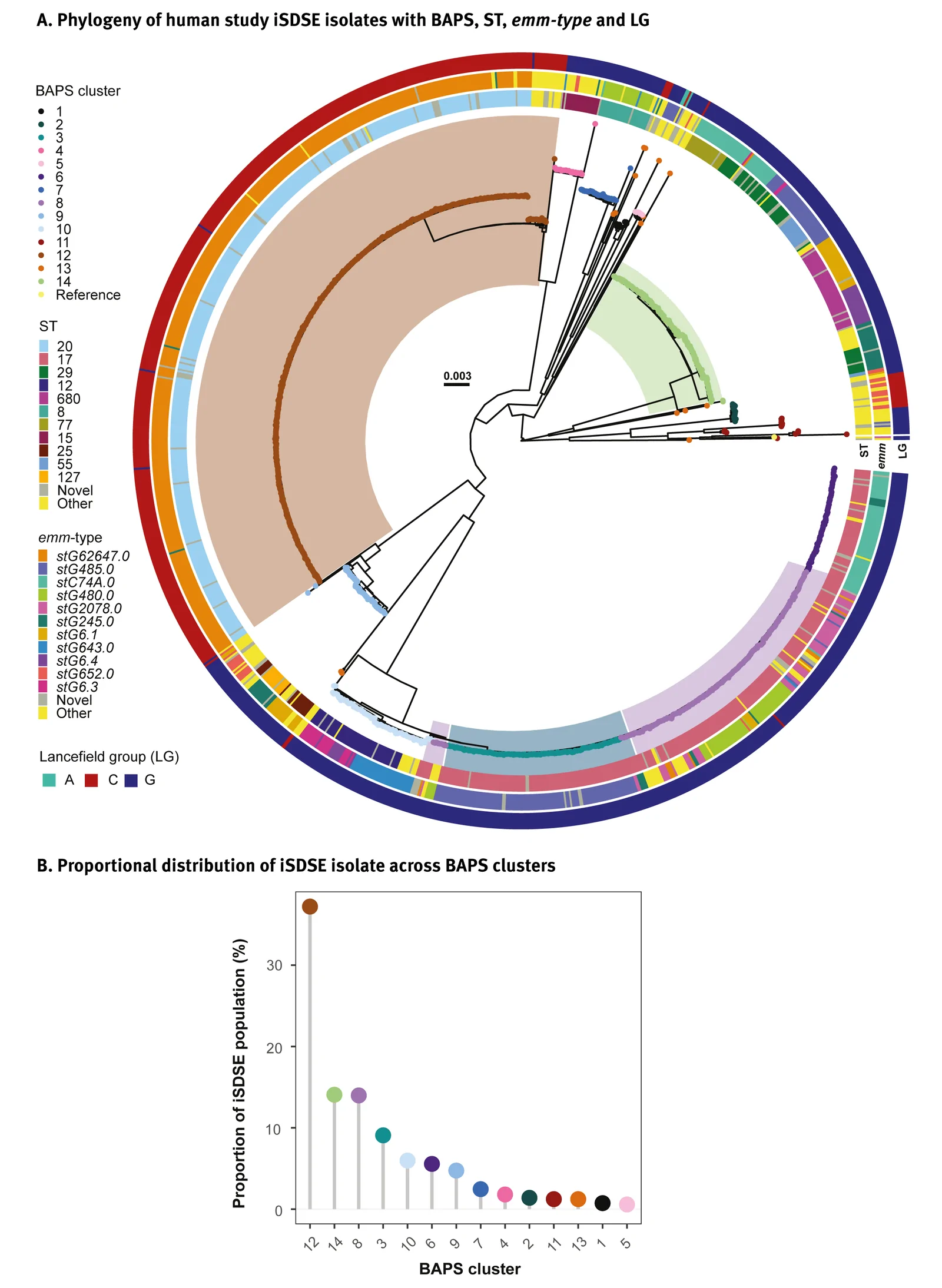
(A) Core genome phylogeny of the iSDSE study population and (B) proportional distribution of iSDSE isolate across BAPS clusters, Denmark (n = 1,218), Greenland (n = 1) and Faroe Islands (n = 4), 17 August 2020–18 September 2022

BAPS: fast hierarchical Bayesian analysis of population structure; iSDSE: invasive *Streptococcus dysgalactiae* subsp. *equisimilis*; LG Lancefield group; SNP: single nucleotide polymorphism; ST: sequence type.

Analysis of the phylogenetic relatedness of all 1,225 study isolates showed two distant isolates (> 10,000 SNPs), as illustrated in Supplementary Figure S1A. By comparing the SNP distances of these two distant isolates with isolates of animal origin identified in a recent Norwegian study [41], we demonstrated clear clustering, indicating an animal origin for the outliers in our sample (Supplementary Figure S1B-C). They were hence excluded from further downstream phylogenetic analyses. This increased the size of the overall conserved core-genome, and thus SNP resolution across the final collection (Figure 3A).

**A.** Midpoint-rooted maximum likelihood phylogeny of the human-related iSDSE study isolates (n = 1,223). The tree is based on 58,305 SNPs using GCF_016128095.1 as the reference. The coloured tips represent the fastBAPS clusters (BAPS). The four dominant BAPS clusters (12, 14, 8 and 3) are identifiable by their colour explained in the BAPS cluster legend. The rings from inner to outer represent ST, *emm*-type and LG, respectively.

**B.** Per cent contribution of each BAPS cluster within the iSDSE human-associated study population (n = 1,223).

Scalebar indicates substitutions per site. The isolates for Denmark arise from 1,218 infection episodes, with two of these episodes involving two genetically distinct iSDSE strains, resulting in a sum of 1,220 isolates; two isolates of animal origin are removed, thus the total number of Danish isolates analysed in the figure is 1,218.

We elicited genetic clusters in the study population (n = 1,223 isolates including those from Denmark, Greenland and the Faroe Islands) using a Bayesian clustering algorithm implemented in fastBAPS [38]. This resulted in 14 Bayesian analysis of population structure (BAPS) clusters. The four most dominant BAPS clusters, 12 (37.2%, n = 455), 14 (14.1%, n = 172), 8 (14.0%, n = 171) and 3 (9.1%, n = 111) altogether represented nearly 75% of the clustered study population (Figure 3B). The most prevalent cluster, cluster 12, correlated well with the dominating ST and *emm*-type, ST20 and *stG62647.0*. Cluster 8, 3 and 6 all had ST17 as their main ST, however they differed by *emm*-type with cluster 3 and 6 comprising mainly of *emm*-type *stG485.0* and *stC74A.0*, respectively, while cluster 8 comprised mainly *stG480.0* and *stG2078.0*.

The most genetically homogenous BAPS clusters were clusters 6, 3, 8 and 12, with median pairwise SNP distance within the cluster of 34, 38, 41 and 60 SNPs, respectively, as depicted in Supplementary Figure S4A. These homogenous genetic clades all had ST20 or ST17 as the dominant ST.

The genetic clusters did not significantly differ by age or sex. We could also not detect a temporal expansion or decline of any of the major BAPS clusters during the study period (Supplementary Figure S4B).

### Phenotypic and genomic antimicrobial resistance

**Figure 4:**
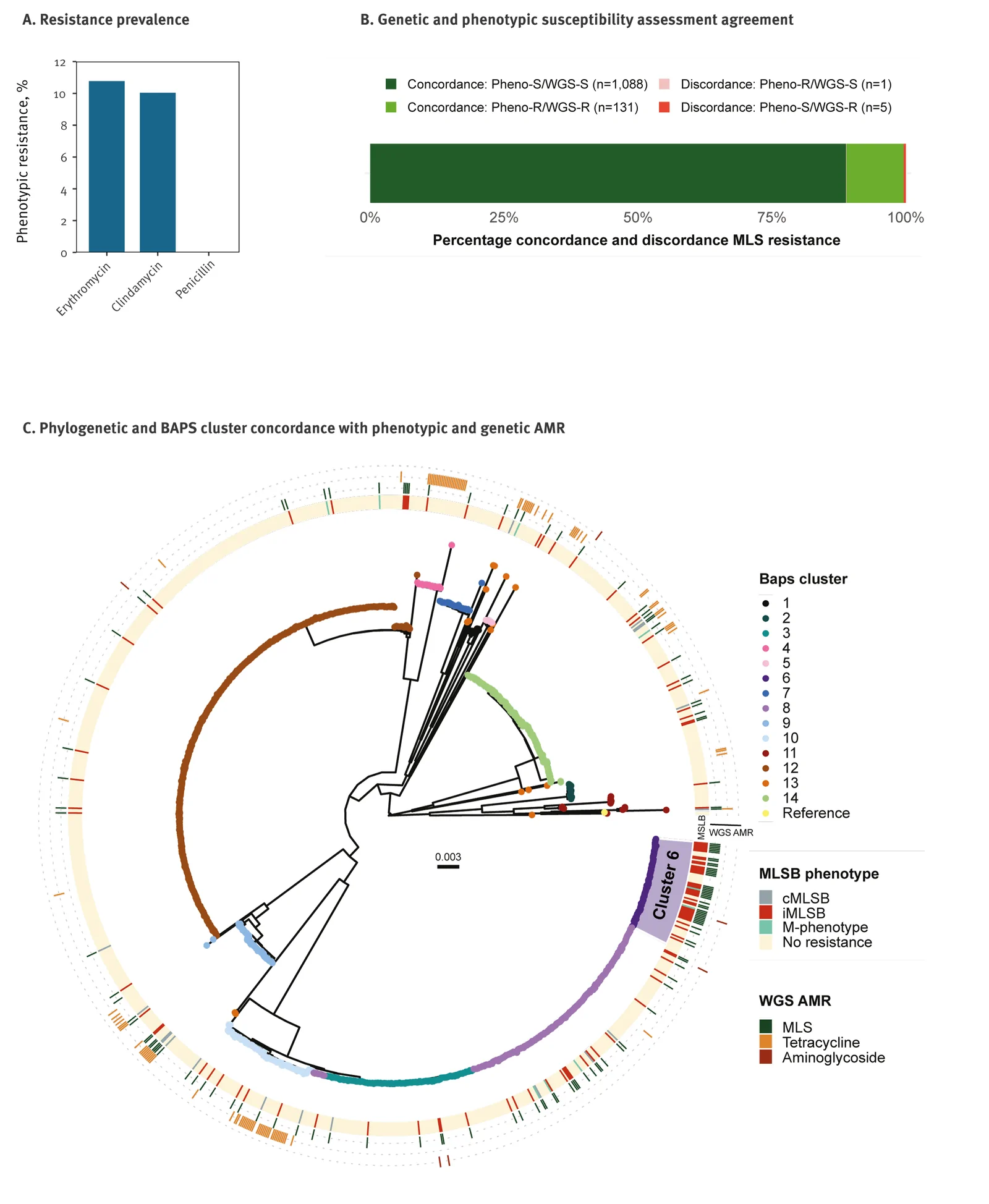
Analysis of the iSDSE study population for (A) phenotypic resistance to erythromycin, clindamycin and penicillin, as well as concordance between (B) phenotypic and genetic MLS susceptibility profiles and (C) BAPS clustering, Denmark (n = 1,220), Greenland (n = 1) and Faroe Islands (n = 4), 17 August 2020−18 September 2022

AMR: antimicrobial resistance; BAPS: fast hierarchical Bayesian analysis of population structure; cMLSB: constitutive MLSB-resistance; iSDSE: invasive *Streptococcus dysgalactiae* subsp. *equisimilis*; iMLSB: inducible MLSB-resistance; M-phenotype: macrolide resistance; MLS: macrolide, lincosamide, and streptogramin; MLSB: macrolide, lincosamide and streptogramin B; pheno-R: phenotypically resistant; pheno-S: phenotypically susceptible; WGS: whole-genome sequencing; WGS-R: suggested resistant by WGS; WGS-S: suggested susceptible by WGS.

Antimicrobial susceptibility testing of the iSDSE isolates showed 100% susceptibility to penicillin. However, we identified per cent resistance prevalences to erythromycin and clindamycin of 10.8% (132/1,225) and 10.0% (123/1,225), respectively (Figure 4A). Of the erythromycin and/or clindamycin resistant isolates, 105 (79.5%) isolates presented an inducible macrolide, lincosamide, and streptogramin B-resistance phenotype (iMLSB), 18 (13.6%) a constitutive MLSB-resistance phenotype (cMLSB), and nine (6.8%) a macrolide-resistant phenotype (M-phenotype), as reported in Supplementary Table S4.

**A.** Phenotypic antimicrobial resistance in the iSDSE study population to erythromycin, clindamycin and penicillin (n = 1,225).

**B.** Concordance and discordance between phenotypic and genotypic resistance to MLS antibiotics (n = 1,225).

**C.** Midpoint rooted maximum likelihood phylogeny of all human-associated iSDSE study isolates (n = 1,223). The tree is based on 58,305 core genome SNPs using GCF_016128095.1 as the reference. The coloured tips represent the fastBAPS (BAPS) clusters. A BAPS cluster (BAPS cluster 6), displaying high MLS resistance prevalence, is highlighted. Inner to outer rings represent MLSB resistance phenotype, followed by presence of antimicrobial resistance genes conferring resistance to MLS, tetracycline and aminoglycosides antibiotic classes. Genes included in the antibiotic classes are listed in a further Table.  Scalebar indicates substitutions per site.

We detected acquired resistance genes in 18.9% (n = 232) of the iSDSE genomes. In total, we detected 13 different AMR genes. The most common ones were associated with macrolide, lincosamide, streptogramin (MLS) and tetracycline resistance. The most frequently detected was *ermA* (9.4%, n = 115), followed by *tet*(M) (5.8%, n = 71) (Table). The MLSB-resistance phenotype correlated well with the detected MLS resistance genes. All but one phenotypically MLSB-resistant isolates carried at least one MLS resistance gene. The majority of the phenotypically MLSB susceptible isolates did not carry any MLS genes (99.5%, 1,088/1,093). Among the 115 *ermA*-positive isolates, the majority (n = 109) presented an MLSB-resistance phenotype, while six *ermA-*positive isolates only displayed an M-phenotype, as outlined by Figure 4B, Supplementary Table S4 and Supplementary Figure S5A.

**Table:** Antimicrobial resistance genes found in iSDSE study population, Denmark (n = 1,220), Greenland (n = 1) and Faroe Islands (n = 4), 17 August 2020–18 September 2022

Antibiotic class	Resistance gene	Count	%
MLS	*ermA*	115	9.4
	*ermB*	15	1.2
	*mefA*	3	0.2
	*msrD*	3	0.2
	*lsaC*	2	0.2
	*ermT*	1	0.1
Tetracycline	*tet*(M)	71	5.8
	*tet*(O)	30	2.4
	*tet*(O/32/O)	6	0.5
	*tet*(S)	2	0.2
	*tet*(W)	1	0.1
Aminoglycoside	*aph3'-III*	6	0.5
	*ant6-Ia*	2	0.2

MLS: macrolide, lincosamide, streptogramin antibiotics.

**Figure 5:**
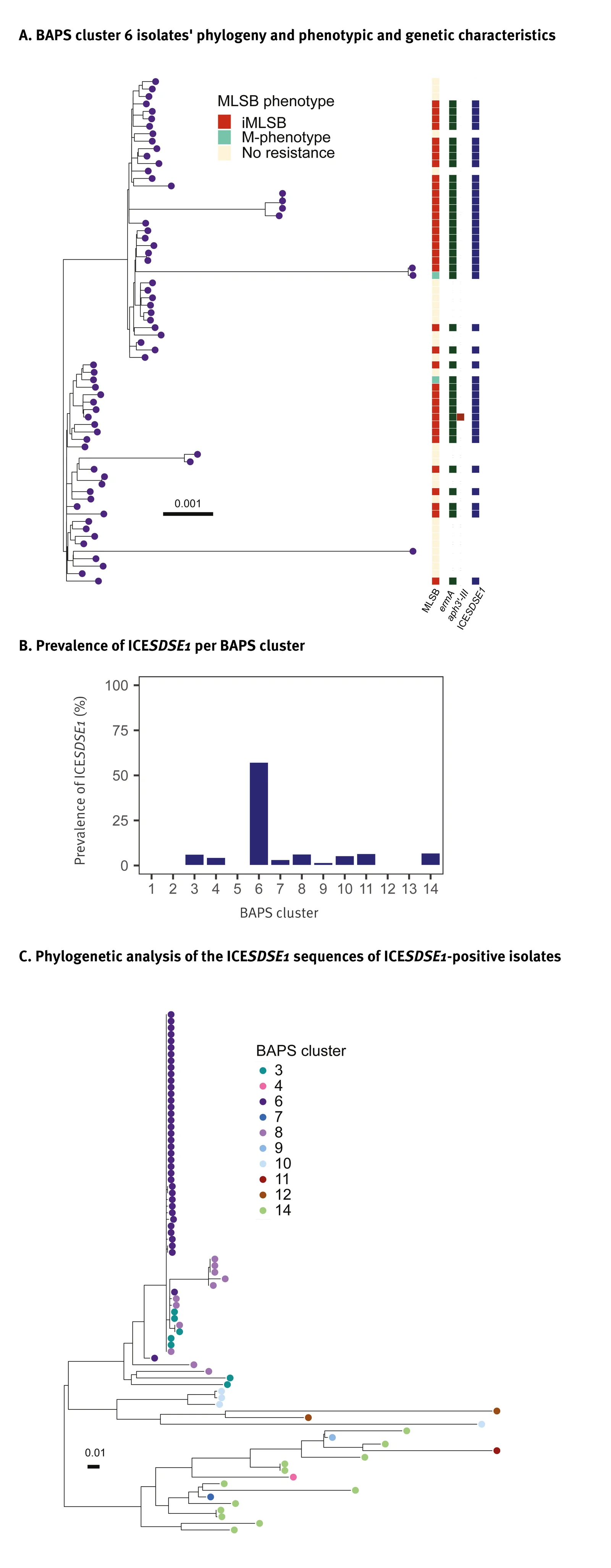
Prevalence of a novel integrative and conjugative element, ICE*SDSE1*, carrying *ermA* among BAPS clusters of study population isolates, Denmark (n = 1,220), Greenland (n = 1) and Faroe Islands (n = 4), 17 August 2020–18 September 2022

BAPS: fast hierarchical Bayesian analysis of population structure; iMLSB: inducible MLSB-resistance; iSDSE: invasive *Streptococcus dysgalactiae* subsp. *equisimilis*; M-phenotype: macrolide resistance; MLSB: macrolide, lincosamide, and streptogramin B resistance.

We investigated if acquired AMR genes were more prevalent in any of the BAPS clusters. We identified that tetracycline resistance genes had a high prevalence in cluster 1 and 4 (77.8% and 100%, respectively). MLS resistance genes had a high prevalence in BAPS cluster 6 (39/68; 57.4%) with *ermA* as the only detected MLS resistant gene (Supplementary Figure S5B). Phenotypic MLSB resistance, iMLSB-phenotype (37/39) and M-phenotype (2/39), in cluster 6 correlated with the presence of *ermA* (Figure 5A). Investigation of the genomic location of *ermA* in the BAPS cluster 6 isolates, showed it as part of a mobile genetic element (MGE), an integrative and conjugative element (ICE) of the ICE family Tn*1549*, henceforth referred to as ICE*SDSE1* (Figure 5A, Supplementary Figure S5C).

**A.** Midpoint-rooted core genome phylogeny of the iSDSE isolates within BAPS cluster 6 (n = 68) based on 13,392 SNPs using GCF_016128095.1 as the reference. The annotations columns from inner to outer represent MLSB-resistance phenotype, presence or absence of *ermA*, *aph3’III*, and ICE*SDSE1*.

**B.** Bar plot depicting the prevalence of ICE*SDSE1* per BAPS cluster. Presence was determined by a k-mer alignment mapping method, with 90% coverage and identity thresholds.

**C**. ICE*SDSE1* phylogeny of ICE*SDSE1*-positive isolates (n = 79) in the study population as identified in (B). The phylogeny is based on 1,298 SNPs using the 34 kb ICE*SDSE1* element from isolate ERS25457729 as reference.

The coloured tips represent the fastBAPS (BAPS) clusters. Scalebar indicates substitutions per site.

We identified that 69% (79/115) of all *ermA* genes in the population were located on this newly described ICE*SDSE1* element, while all (n = 39) of the cluster 6 *ermA* genes were located on ICE*SDSE1*. In all other BAPS clusters, the presence of ICE*SDSE1* was below 7% (Figure 5B). All detected ICE*SDSE1* in the study population carried *ermA*. Analysis of ICE*SDSE1* belonging to BAPS cluster 6 showed that these were genetically highly homogenous, suggesting a recent acquisition. ICE*SDSE1* displayed a higher genetic diversity in isolates belonging to other BAPS clusters (Figure 5C, Supplementary Figure S5D).

## Discussion

Our study estimates for the first time iSDSE incidence-rates using registry data in Denmark. The iSDSE incidence rates increased significantly in Denmark over the 11-year study period between 2014 and 2024, peaking in 2023 with 16.4 per 100,000 individuals. Our findings support a recent study that reported an increasing trend in incidence of invasive GCS (iGCS) and GGS infections using national laboratory-surveillance data in Denmark between 2012 and 2023. The study reported an increase for iGCS from 1.4 to 4.8 per 100,000 and for iGGS from 3.1 to 8.5 per 100,000 between 2012 and 2023 [14]. However, the estimated incidence rates reported in the study were based on voluntarily submitted isolates through the national laboratory surveillance system and not on registry data and thereby likely underestimating the incidence rate. Further, reporting by serogroups, i.e. GCS and GGS, will comprise SDSE and also other streptococcal species, which can modify the trend if the change is species dependent.

Registry-based studies on the incidence of iSDSE remain limited, often restricted geographically or regionally within a country. Comparing incidence rates between countries is further challenged by classifying and/or separating infections by Lancefield group, i.e. GCS, GGS, and Group C and G streptococci (GCGS), instead of reporting infections by species. Worldwide, incidence rates of invasive SDSE or GCGS infections have been reported to be on the increase [5,8-11,17]. Nordic countries other than Denmark, i.e. Norway and Finland, have described increased bSDSE incidence rates [5,15,19]. In Western Norway incidence rates of bSDSE infection have risen from 1.4 to 7.6 per 100,000 inhabitants during 1999–2021 [17]. In Finland, incidence rates have also been increasing in the Pirkanmaa area since the mid-1990s [5], and the mean annual incidence rate of bSDSE for the Pirkanmaa health district was 16.9 per 100,000 inhabitants during the period covering 2015 to 2018 [15]. National invasive GCGS incidence rates in Finland showed an increasing trend between 2006 and 2020 with an annual incidence rate of 17.6 per 100,000 in 2020 [16]. In both Finland and in Denmark GCGS is the most common beta-haemolytic streptococcus causing invasive infection surpassing Group A *Streptococcus* (GAS) and Group B *Streptococcus* (GBS) [14,16]. Our reported incidence rate for both invasive SDSE infections and SDSE bacteraemia i, ranging between 10.3 to 16.4 and 6.9 to 12.4 per 100,000 inhabitants, respectively, in Denmark, remained lower than those reported in Finland during the corresponding years. 

In spring 2020, Denmark, like many countries worldwide, introduced COVID-19 pandemic restrictions. Unlike reports for *S. pyogenes* in Denmark [14,47], we did not observe a decrease in iSDSE incidence rates during this period. This suggests that the transmission dynamics of *S. dysgalactiae* and *S. pyogenes* differ substantially, a conclusion further supported by the relatively stable cluster-distribution of iSDSE during the COVID-19 epidemic observed in our study. Furthermore, we found that older adults and males were most affected by iSDSE infection, consistent with previous reports [15]. Collectively, our findings support the need for a registry-based surveillance system in Denmark to further assess the situation in the future and that invasive SDSE or GCGS infection require more attention.

The genomic insight provided from our study, gives the first comprehensive overview of the genomic diversity within the Danish iSDSE isolate population during the 2-year period (Aug 2020–Sep 2022) sequencing was performed. Genetic clustering showed that the population is dominated by four clusters comprising 75% of the population. The remaining population showed a higher genomic diversity. We detected two dominant STs in the isolate population, ST17 and ST20, which both dominate in Europe [30,48]. The most prevalent *emm*-type in Denmark during the study period was *stG62647*, previously associated with higher virulence [49] is also the predominant *emm*-type in several European countries and Canada [17,31,48,50].

Analyses detected two isolates that clustered with animal-origin strains, which further highlights the zoonotic potential of SDSE [41,51]. Penicillin-resistant SDSE are rarely described in humans [20]. However, a recently published study that investigated slaughtered pigs in India reported that all isolated SDSE strains were resistant to penicillin, to erythromycin and to clindamycin [52]. We did not detect any SDSE isolates resistant to penicillin, which is the first line treatment for SDSE infection. Clindamycin resistance was 10.0% (123/1225), of which the majority displayed (105/123) the inducible clindamycin resistance phenotype. In our study population, resistance to erythromycin was observed in 10.8% of isolates, comparable to rates documented for Norway [21,51], yet lower than the rate in a study from Taiwan [22]. The proportion in our study was also less than rates reported from various Asian countries, e.g. for Japan, South Korea, and China [22-24]. Resistance to second-line treatment options (macrolides and lincosamides) can pose a challenge in treatment options for patients with beta-lactam allergies. The concordance between phenotypic and genomic MLS resistance was high and genomic prediction could potentially be an important part of AMR surveillance in Denmark. Analyses revealed that 69% of all *ermA* genes were located on a novel ICE belonging to the ICE Tn*1549* family, named ICE*SDSE1,* which was predominantly found in cluster 6. Within cluster 6, ICE*SDSE1* showed greater genetic homogeneity compared with other clusters, suggesting recent integration. Conjugative transfer of MGEs facilitates rapid dissemination of AMR [53] and has been proposed as an important mechanism in beta-haemolytic streptococci [21]. Recent evidence also supports cross-species exchange of near-identical MGEs carrying AMR genes between SDSE and *S. pyogenes* [54]. Our results further underscore the significance of MGEs for AMR gene dissemination in SDSE.

Our study contains a number of limitations. The registry-based incidence rates are based on MiBa extracts that can include group C or group G streptococci that are not SDSE. We may lack Lancefield group A or L SDSE cases, if they were reported not as *Streptococcus dysgalactiae* but rather by their Lancefield group. Further, our definition of an invasive case is solely laboratory-based by sample location and type without clinical criteria. We did not have access to information about any underlying diseases among the patients with iSDSE, nor about the outcome of the infection. Still, our study is the first nationwide register-based assessment of iSDSE incidence, independent of voluntary isolates submissions from regional laboratories. Our genomic study cohort spans around 2 years and is therefore not suited to investigate longer temporal patterns. Further, our genomic study population only contains isolates that were voluntarily submitted to SSI and therefore does not provide a comprehensive collection of all iSDSE strains in the country. It is, however, one of the largest iSDSE surveillance datasets published so far and the first genomic-based surveillance dataset from Denmark.

## Conclusion

Our findings provide the first registry-based estimate of iSDSE incidence in Denmark, demonstrating a significant increase over the past decade. Routine registry-based surveillance would be valuable for monitoring this trend and potentially detecting outbreaks. Further, molecular epidemiology findings provided valuable insights into the genetic composition of the iSDSE population in Denmark, highlighting key variants, population genomic diversity, and the prevalence of phenotypic and genomic antimicrobial resistance. Although no resistance to the first-line treatment, penicillin, was detected, resistance to the second-line drug erythromycin was relatively common, especially in one genetic cluster. This finding is of concern, especially for the treatment of patients with beta-lactam allergy, and emphasises the importance of continued surveillance of anitimicrobial resistance patterns in iSDSE.

## Data Availability

The genomic sequence data for the 1,225 study isolates that passed quality control have been deposited at the European Nucleotide Archive (ENA) under BioProject accession ID PRJEB95956.

## Note

Jana Grüttner is a fellow of the ECDC Fellowship Programme, supported financially by the European Centre for Disease Prevention and Control. The views and opinions expressed herein do not state or reflect those of ECDC. ECDC is not responsible for the data and information collation and analysis and cannot be held liable for conclusions or opinions drawn.

## Supplementary Data

6007000 Supplementary Tables and Figures

## Supplementary Data

24000 Supplementary Data

## References

[1] Lancefield RC. A Serological Differentiation Of Human And Other Groups Of Hemolytic Streptococci. J Exp Med. 1933;57(4):571-95. 10.1084/jem.57.4.571 19870148 PMC2132252

[2] Brandt CM Spellerberg B. Human infections due to Streptococcus dysgalactiae subspecies equisimilis. Clin Infect Dis. 2009;49(5):766-72. 10.1086/605085 19635028

[3] Chochua S Rivers J Mathis S Li Z Velusamy S McGee L Emergent Invasive Group A Streptococcus dysgalactiae subsp. equisimilis, United States, 2015-2018. Emerg Infect Dis. 2019;25(8):1543-7. 10.3201/eid2508.181758 31158071 PMC6649341

[4] Hashikawa S Iinuma Y Furushita M Ohkura T Nada T Torii K Characterization of group C and G streptococcal strains that cause streptococcal toxic shock syndrome. J Clin Microbiol. 2004;42(1):186-92. 10.1128/JCM.42.1.186-192.2004 14715751 PMC321656

[5] Rantala S Vuopio-Varkila J Vuento R Huhtala H Syrjänen J. Clinical presentations and epidemiology of β-haemolytic streptococcal bacteraemia: a population-based study. Clin Microbiol Infect. 2009;15(3):286-8. 10.1111/j.1469-0691.2008.02672.x 19175622

[6] Bruun T Kittang BR de Hoog BJ Aardal S Flaatten HK Langeland N Necrotizing soft tissue infections caused by Streptococcus pyogenes and Streptococcus dysgalactiae subsp. equisimilis of groups C and G in western Norway. Clin Microbiol Infect. 2013;19(12):E545-50. 10.1111/1469-0691.12276 23795951

[7] Liao CH Liu LC Huang YT Teng LJ Hsueh PR. Bacteremia caused by group G Streptococci, taiwan. Emerg Infect Dis. 2008;14(5):837-40. 10.3201/eid1405.070130 18439377 PMC2600252

[8] Schwartz IS Keynan Y Gilmour MW Dufault B Lagacé-Wiens P. Changing trends in β-hemolytic streptococcal bacteremia in Manitoba, Canada: 2007-2012. Int J Infect Dis. 2014;28:211-3. 10.1016/j.ijid.2014.03.1376 25403914

[9] Sylvetsky N Raveh D Schlesinger Y Rudensky B Yinnon AM. Bacteremia due to beta-hemolytic Streptococcus group G: increasing incidence and clinical characteristics of patients. Am J Med. 2002;112(8):622-6. 10.1016/S0002-9343(02)01117-8 12034411

[10] Shinohara K Murase K Tsuchido Y Noguchi T Yukawa S Yamamoto M Clonal Expansion of Multidrug-Resistant Streptococcus dysgalactiae Subspecies equisimilis Causing Bacteremia, Japan, 2005-2021. Emerg Infect Dis. 2023;29(3):528-39. 10.3201/eid2903.221060 36823027 PMC9973691

[11] Gajdács M Ábrók M Lázár A Burián K. Beta-haemolytic group A, C and G streptococcal infections in southern hungary: A 10-year population-based retrospective survey (2008–2017) and a review of the literature. Infect Drug Resist. 2020;13:4739-49. 10.2147/IDR.S279157 33408489 PMC7781025

[12] Ekelund K Skinhøj P Madsen J Konradsen HB. Invasive group A, B, C and G streptococcal infections in Denmark 1999-2002: epidemiological and clinical aspects. Clin Microbiol Infect. 2005;11(7):569-76. 10.1111/j.1469-0691.2005.01169.x 15966976

[13] Lambertsen LM Ingels H Schønheyder HC Hoffmann S Danish Streptococcal Surveillance Collaboration Group 2011. Nationwide laboratory-based surveillance of invasive beta-haemolytic streptococci in Denmark from 2005 to 2011. Clin Microbiol Infect. 2014;20(4):O216-23. 10.1111/1469-0691.12378 24125634 PMC4232002

[14] Christiansen CH Søgaard KK Dam-Dalgeir G Dessau RB Dzajic E Jensen CS Surveillance of invasive beta-haemolytic streptococci in Denmark, 2012 to 2023: A nationwide study. J Infect. 2025;91(2):106559. 10.1016/j.jinf.2025.106559 40714117

[15] Nevanlinna V Huttunen R Aittoniemi J Luukkaala T Rantala S. Incidence, seasonal pattern, and clinical manifestations of Streptococcus dysgalactiae subspecies equisimilis bacteremia; a population-based study. Eur J Clin Microbiol Infect Dis. 2023;42(7):819-25. 10.1007/s10096-023-04607-8 37119347 PMC10267002

[16] Paspaliari DK Sarvikivi E Ollgren J Vuopio J. Invasive beta-haemolytic streptococcal infections, Finland, 2006 to 2020: increase in Lancefield group C/G infections. Euro Surveill. 2023;28(31):2200807. 10.2807/1560-7917.ES.2023.28.31.2200807 37535473 PMC10401913

[17] Oppegaard O Glambek M Skutlaberg DH Skrede S Sivertsen A Kittang BR. Streptococcus dysgalactiae Bloodstream Infections, Norway, 1999-2021. Emerg Infect Dis. 2023;29(2):260-7. 10.3201/eid2902.221218 36692331 PMC9881787

[18] Baracco GJ. Infections Caused by Group C and G Streptococcus (Streptococcus dysgalactiae subsp. equisimilis and Others): Epidemiological and Clinical Aspects. Microbiol Spectr. 2019;7(2):7.2.32. 30977463 10.1128/microbiolspec.gpp3-0016-2018 PMC11590429

[19] Rantala S. Streptococcus dysgalactiae subsp. equisimilis bacteremia: an emerging infection. Eur J Clin Microbiol Infect Dis. 2014;33(8):1303-10. 10.1007/s10096-014-2092-0 24682845

[20] Fuursted K Stegger M Hoffmann S Lambertsen L Andersen PS Deleuran M Description and characterization of a penicillin-resistant Streptococcus dysgalactiae subsp. equisimilis clone isolated from blood in three epidemiologically linked patients. J Antimicrob Chemother. 2016;71(12):3376-80. 10.1093/jac/dkw320 27585966

[21] Oppegaard O Skrede S Mylvaganam H Kittang BR. Emerging Threat of Antimicrobial Resistance in β-Hemolytic Streptococci. Front Microbiol. 2020;11:797. 10.3389/fmicb.2020.00797 32477287 PMC7242567

[22] Lo HH Nien HH Cheng YY Su FY. Antibiotic susceptibility pattern and erythromycin resistance mechanisms in beta-hemolytic group G Streptococcus dysgalactiae subspecies equisimilis isolates from central Taiwan. J Microbiol Immunol Infect. 2015;48(6):613-7. 10.1016/j.jmii.2014.04.003 24856419

[23] Kim S Byun JH Park H Lee J Lee HS Yoshida H Molecular Epidemiological Features and Antibiotic Susceptibility Patterns of Streptococcus dysgalactiae subsp. equisimilis Isolates from Korea and Japan. Ann Lab Med. 2018;38(3):212-9. 10.3343/alm.2018.38.3.212 29401555 PMC5820065

[24] Lu B Fang Y Huang L Diao B Du X Kan B Molecular characterization and antibiotic resistance of clinical Streptococcus dysgalactiae subsp. equisimilis in Beijing, China. Infect Genet Evol. 2016;40:119-25. 10.1016/j.meegid.2016.01.030 26925701

[25] Jolley KA Bray JE Maiden MCJ. Open-access bacterial population genomics: BIGSdb software, the PubMLST.org website and their applications. Wellcome Open Res. 2018;3:124. 10.12688/wellcomeopenres.14826.1 30345391 PMC6192448

[26] McMillan DJ Bessen DE Pinho M Ford C Hall GS Melo-Cristino J Population genetics of Streptococcus dysgalactiae subspecies equisimilis reveals widely dispersed clones and extensive recombination. PLoS One. 2010;5(7):e11741. 10.1371/journal.pone.0011741 20668530 PMC2909212

[27] Kittang BR Langeland N Mylvaganam H. Distribution of emm types and subtypes among noninvasive group A, C and G streptococcal isolates in western Norway. APMIS. 2008;116(6):457-64. 10.1111/j.1600-0463.2008.00976.x 18754319

[28] Leitner E Zollner-Schwetz I Zarfel G Masoud-Landgraf L Gehrer M Wagner-Eibel U Prevalence of emm types and antimicrobial susceptibility of Streptococcus dysgalactiae subsp. equisimilis in Austria. Int J Med Microbiol. 2015;305(8):918-24. 10.1016/j.ijmm.2015.10.001 26507866

[29] Pinho MD Melo-Cristino J Ramirez M. Clonal relationships between invasive and noninvasive Lancefield group C and G streptococci and emm-specific differences in invasiveness. J Clin Microbiol. 2006;44(3):841-6. 10.1128/JCM.44.3.841-846.2006 16517864 PMC1393098

[30] Kaci A Jonassen CM Skrede S Sivertsen A Steinbakk M Oppegaard O Norwegian Study Group on Streptococcus dysgalactiae. Genomic epidemiology of Streptococcus dysgalactiae subsp. *equisimilis* strains causing invasive disease in Norway during 2018. Front Microbiol. 2023;14:1171913. 10.3389/fmicb.2023.1171913 37485526 PMC10361778

[31] Lother SA Demczuk W Martin I Mulvey M Dufault B Lagacé-Wiens P Clonal Clusters and Virulence Factors of Group C and G Streptococcus Causing Severe Infections, Manitoba, Canada, 2012-2014. Emerg Infect Dis. 2017;23(7):1079-88. 10.3201/eid2307.161259 28628457 PMC5512470

[32] Voldstedlund M Haarh M Mølbak K MiBa Board of Representatives. MiBa Board of Representatives. The danish microbiology database (MIBA) 2010 to 2013. Euro Surveill. 2014;19(1):20667. 10.2807/1560-7917.ES2014.19.1.20667 24434175

[33] Holm S. A simple sequentially rejective multiple test procedure. Scand J Stat Theory Appl. 1979;6(2):65-70. Available from: https://www.jstor.org/stable/4615733

[34] R Core Team. R: A Language and Environment for Statistical Computing. Vienna: R Foundation for Statistical Computing; 2023. Available from: https://www.r-project.org/

[35] Souvorov A Agarwala R Lipman DJ. SKESA: strategic k-mer extension for scrupulous assemblies. Genome Biol. 2018;19(1):153. 10.1186/s13059-018-1540-z 30286803 PMC6172800

[36] Sahl JW Lemmer D Travis J Schupp JM Gillece JD Aziz M NASP: an accurate, rapid method for the identification of SNPs in WGS datasets that supports flexible input and output formats. Microb Genom. 2016;2(8):e000074. 10.1099/mgen.0.000074 28348869 PMC5320593

[37] Croucher NJ Page AJ Connor TR Delaney AJ Keane JA Bentley SD Rapid phylogenetic analysis of large samples of recombinant bacterial whole genome sequences using Gubbins. Nucleic Acids Res. 2015;43(3):e15-15. 10.1093/nar/gku1196 25414349 PMC4330336

[38] Tonkin-Hill G Lees JA Bentley SD Frost SDW Corander J. Fast hierarchical Bayesian analysis of population structure. Nucleic Acids Res. 2019;47(11):5539-49. 10.1093/nar/gkz361 31076776 PMC6582336

[39] Minh BQ Schmidt HA Chernomor O Schrempf D Woodhams MD von Haeseler A IQ-TREE 2: New Models and Efficient Methods for Phylogenetic Inference in the Genomic Era. Mol Biol Evol. 2020;37(5):1530-4. 10.1093/molbev/msaa015 32011700 PMC7182206

[40] Price MN Dehal PS Arkin AP. FastTree 2--approximately maximum-likelihood trees for large alignments. PLoS One. 2010;5(3):e9490. 10.1371/journal.pone.0009490 20224823 PMC2835736

[41] Porcellato D Smistad M Skeie SB Jørgensen HJ Austbø L Oppegaard O. Whole genome sequencing reveals possible host species adaptation of Streptococcus dysgalactiae. Sci Rep. 2021;11(1):17350. 10.1038/s41598-021-96710-z 34462475 PMC8405622

[42] Clausen PTLC Aarestrup FM Lund O. Rapid and precise alignment of raw reads against redundant databases with KMA. BMC Bioinformatics. 2018;19(1):307. 10.1186/s12859-018-2336-6 30157759 PMC6116485

[43] Bortolaia V Kaas RS Ruppe E Roberts MC Schwarz S Cattoir V ResFinder 4.0 for predictions of phenotypes from genotypes. J Antimicrob Chemother. 2020;75(12):3491-500. 10.1093/jac/dkaa345 32780112 PMC7662176

[44] Lao J Lacroix T Guédon G Coluzzi C Payot S Leblond-Bourget N ICEscreen: a tool to detect Firmicute ICEs and IMEs, isolated or enclosed in composite structures. NAR Genom Bioinform. 2022;4(4):lqac079. 10.1093/nargab/lqac079 36285285 PMC9585547

[45] Wang M, Liu G, Liu M, Tai C, Deng Z, Song J, et al. ICEberg 3.0: functional categorization and analysis of the integrative and conjugative elements in bacteria. Nucleic Acids Res. 2024;52(D1) no. D1;D732-7. 37870467 10.1093/nar/gkad935 PMC10767825

[46] Prjibelski A Antipov D Meleshko D Lapidus A Korobeynikov A. Using SPAdes De Novo Assembler. Curr Protoc Bioinformatics. 2020;70(1):e102. 10.1002/cpbi.102 32559359

[47] Johannesen TB Munkstrup C Edslev SM Baig S Nielsen S Funk T Increase in invasive group A streptococcal infections and emergence of novel, rapidly expanding sub-lineage of the virulent Streptococcus pyogenes M1 clone, Denmark, 2023. Euro Surveill. 2023;28(26):2300291. 10.2807/1560-7917.ES.2023.28.26.2300291 37382884 PMC10311951

[48] López de Egea G González-Díaz A Olsen RJ Guédon G Berbel D Grau I Emergence of invasive Streptococcus dysgalactiae subsp. equisimilis in Spain (2012-2022): genomic insights and clinical correlations. Int J Infect Dis. 2025;153:107778. 10.1016/j.ijid.2025.107778 39800082

[49] Oppegaard O Mylvaganam H Skrede S Lindemann PC Kittang BR. Emergence of a Streptococcus dysgalactiae subspecies equisimilis stG62647-lineage associated with severe clinical manifestations. Sci Rep. 2017;7(1):7589. 10.1038/s41598-017-08162-z 28790435 PMC5548910

[50] Itzek A Weißbach V Meintrup D Rieß B van der Linden M Borgmann S. Epidemiological and Clinical Features of Streptococcus dysgalactiae ssp. equisimilis stG62647 and Other emm Types in Germany. Pathogens. 2023;12(4):589. 10.3390/pathogens12040589 37111475 PMC10143538

[51] Glambek M Skrede S Sivertsen A Kittang BR Kaci A Jonassen CM Antimicrobial resistance patterns in Streptococcus dysgalactiae in a One Health perspective. Front Microbiol. 2024;15:1423762. 10.3389/fmicb.2024.1423762 39193432 PMC11348040

[52] Patel SM Sahoo M Thakor JC Murali D Kumar P Singh R Pathomolecular epidemiology, antimicrobial resistance, and virulence genes of Streptococcus dysgalactiae subsp. equisimilis isolates from slaughtered pigs in India. J Appl Microbiol. 2024;135(1):1-11. 10.1093/jambio/lxae002 38178631

[53] Partridge SR Kwong SM Firth N Jensen SO. Mobile genetic elements associated with antimicrobial resistance. Clin Microbiol Rev. 2018;31(4):e00088-17. 10.1128/CMR.00088-17 30068738 PMC6148190

[54] Xie O Zachreson C Tonkin-Hill G Price DJ Lacey JA Morris JM Overlapping Streptococcus pyogenes and Streptococcus dysgalactiae subspecies equisimilis household transmission and mobile genetic element exchange. Nat Commun. 2024;15(1):3477. 10.1038/s41467-024-47816-1 38658529 PMC11043366

