# Supplementary material for: Emerging trends in invasive Streptococcus dysgalactiae subsp. equisimilis infections in Denmark, 2014 to 2024: a nationwide genomic and registry-based study
Source: Euro Surveill. 2026 Sep 3;31(35):2500714. doi: 10.2807/1560-7917.ES.2026.31.35.2500714 (PMC13545521; doi:10.2807/1560-7917.ES.2026.31.35.2500714)
Supplement: Supplementary file 1 [file 25-00714_STEGGER_Supplementary_Tables_Figures.pdf]

This supplementary material is hosted by Eurosurveillance as supporting information alongside the article "Emerging trends in invasive *Streptococcus dysgalactiae* subsp. *equisimilis* infections in Denmark, 2014 to 2024: a nationwide genomic and registry-based study", on behalf of the authors, who remain responsible for the accuracy and appropriateness of the content. The same standards for ethics, copyright, attributions and permissions as for the article apply. Supplements are not edited by Eurosurveillance and the journal is not responsible for the maintenance of any links or email addresses provided therein.

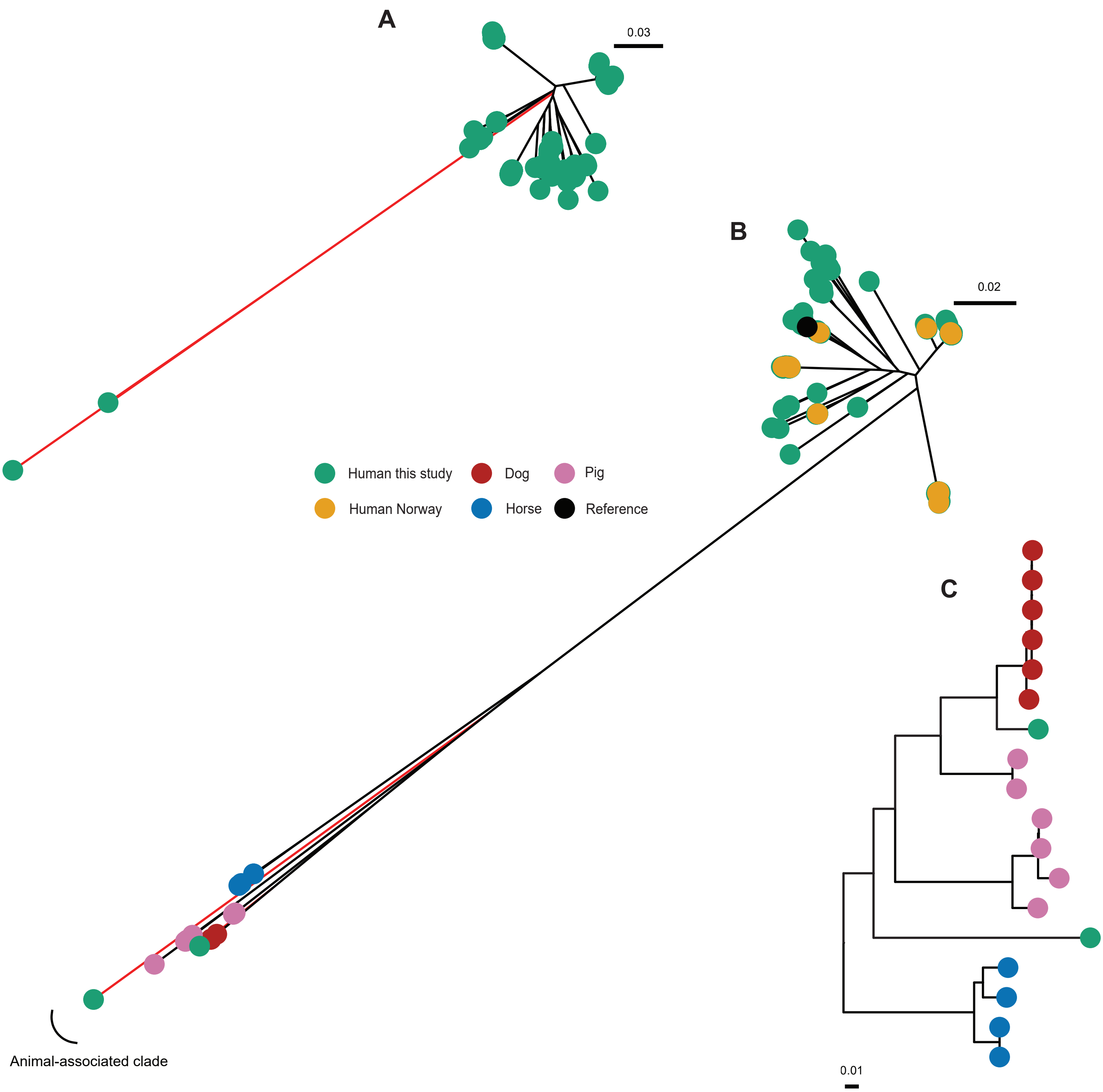


**Supplementary Figure S1. (A)** Unrooted core genome phylogeny of iSDSE study isolates (n=1,225). The mid-point rooted phylogenetic tree is based on 78,369 SNPs using GCF_016128095.1 as the reference. **(B)** Unrooted core genome phylogeny of the study population and human and animal SDSE isolates from Norway from the study of Porcellato *et al.*, 2021 (n= 1,266). The tree is based on 89,008 SNPs using GCF_016128095.1 as the reference. Red tree branches indicate the two iSDSE outlier isolates from the study population in panel (A) and (B). **(C)** Detailed view of the animal-associated clade from panel B. Coloured tips represent the isolate's host origin across all panels. Scalebars indicate substitutions per site.

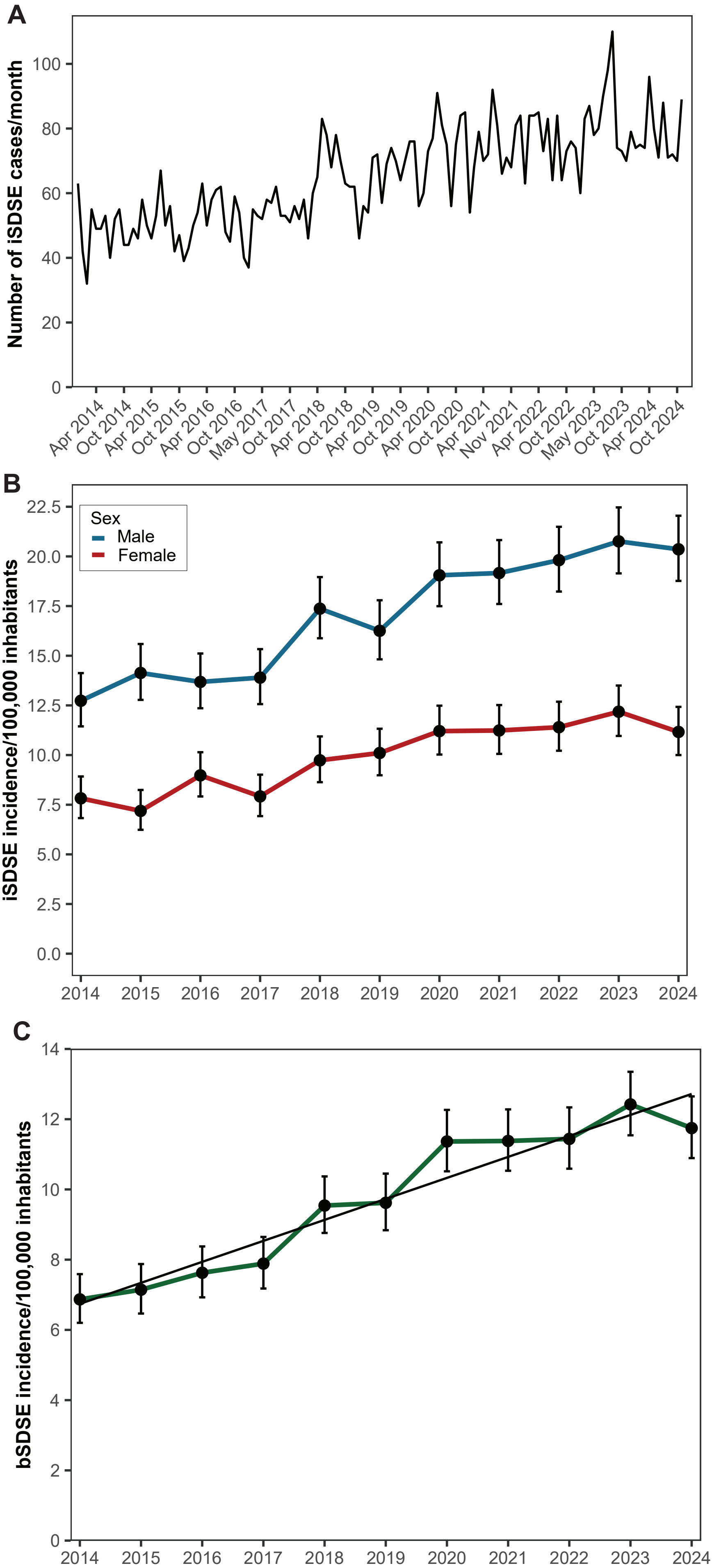


**Supplementary Figure S2. (A)** Monthly iSDSE cases in Denmark 2014-2024 (n= 8,603). **(B)** Mean annual incidence rate per 100,000 of iSDSE infections for males (blue) and females (red) in Denmark between 2014-2024. **(C)** Mean annual incidence rates per 100,000 of bacteraemia SDSE (bSDSE) infections in Denmark 2014-2024 (n= 6,232). A linear regression trend line is indicated in black.

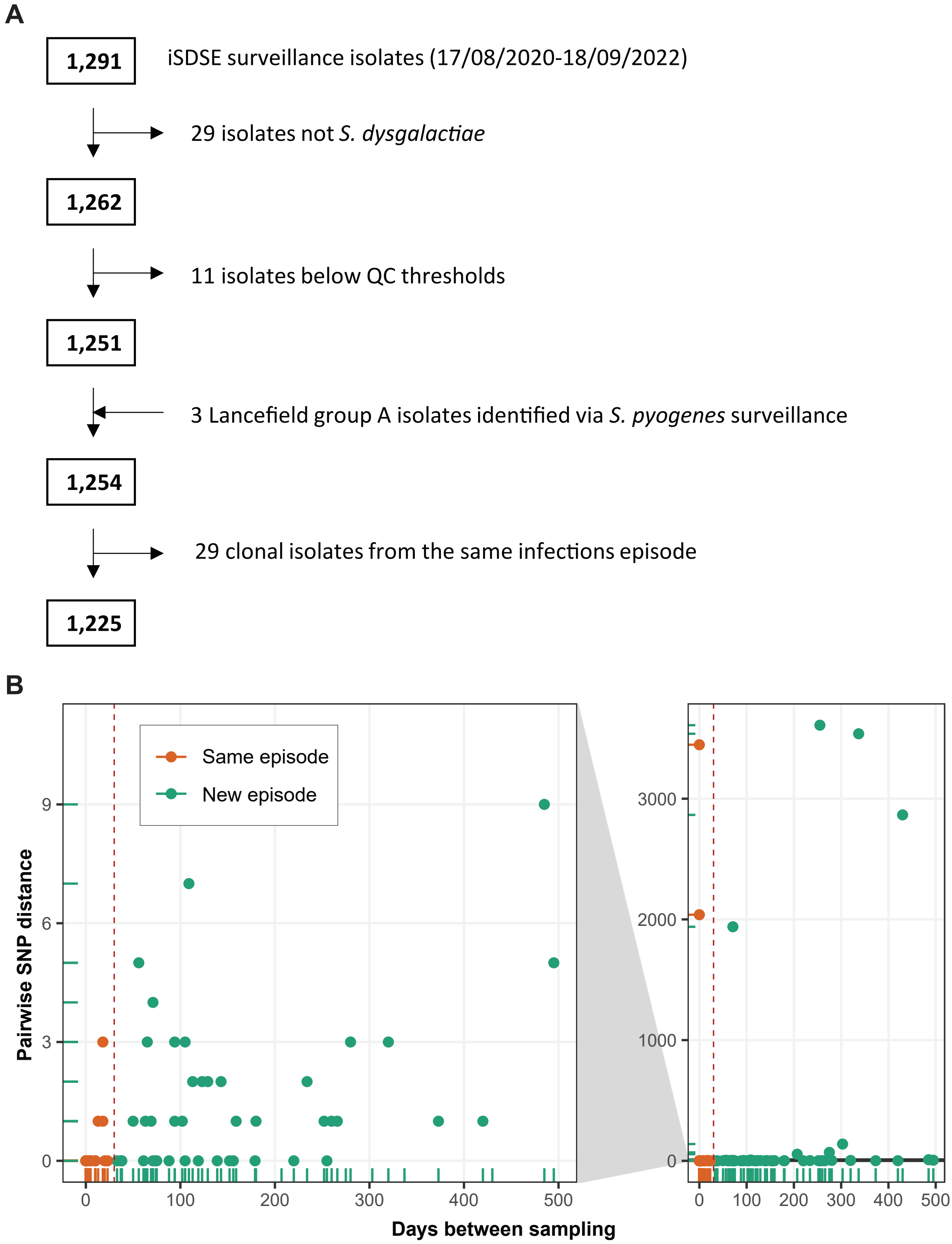


**Supplementary Figure S3. (A)** Illustration of the isolate selection procedure for the genomic study cohort with a final isolate count of 1,225. **(B)** Pairwise SNP distances after purging for recombination between concurrent samples from the same individual. An infection episode definition of a 30-day period after the last identified isolate from the same individual is indicated by colour (orange, same episode; green, new episode). The left panel is a zoom in between 1-12 pairwise SNPs. SNP, Single nucleotide polymorphism.

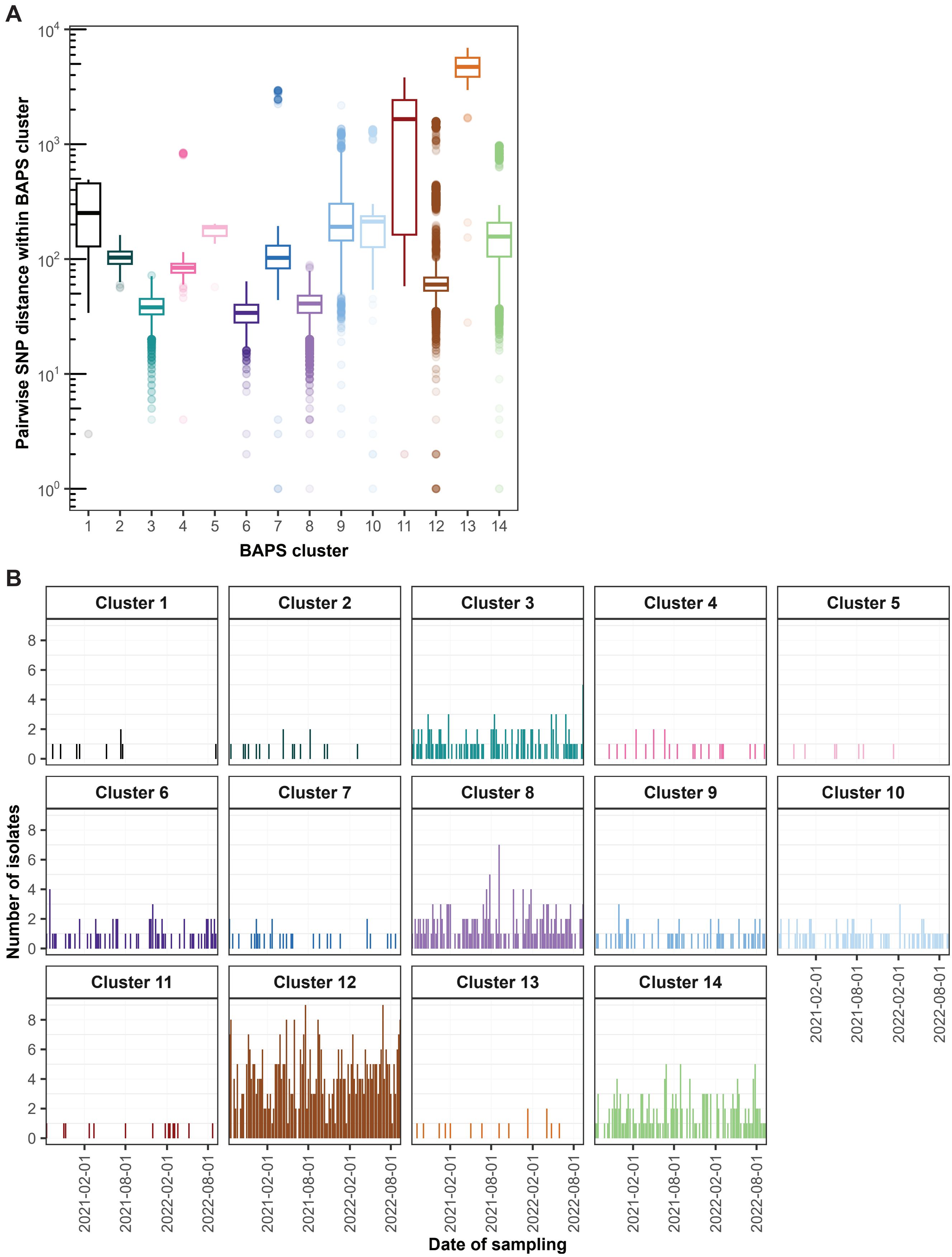


**Supplementary Figure S4. (A)** Pairwise SNP distances between iSDSE study isolates (n=1,223) within a BAPS cluster. **(B)** Weekly counts of iSDSE study isolates (n=1,223) per BAPS cluster plotted over the study period (17/08/2020-18/09/2022).

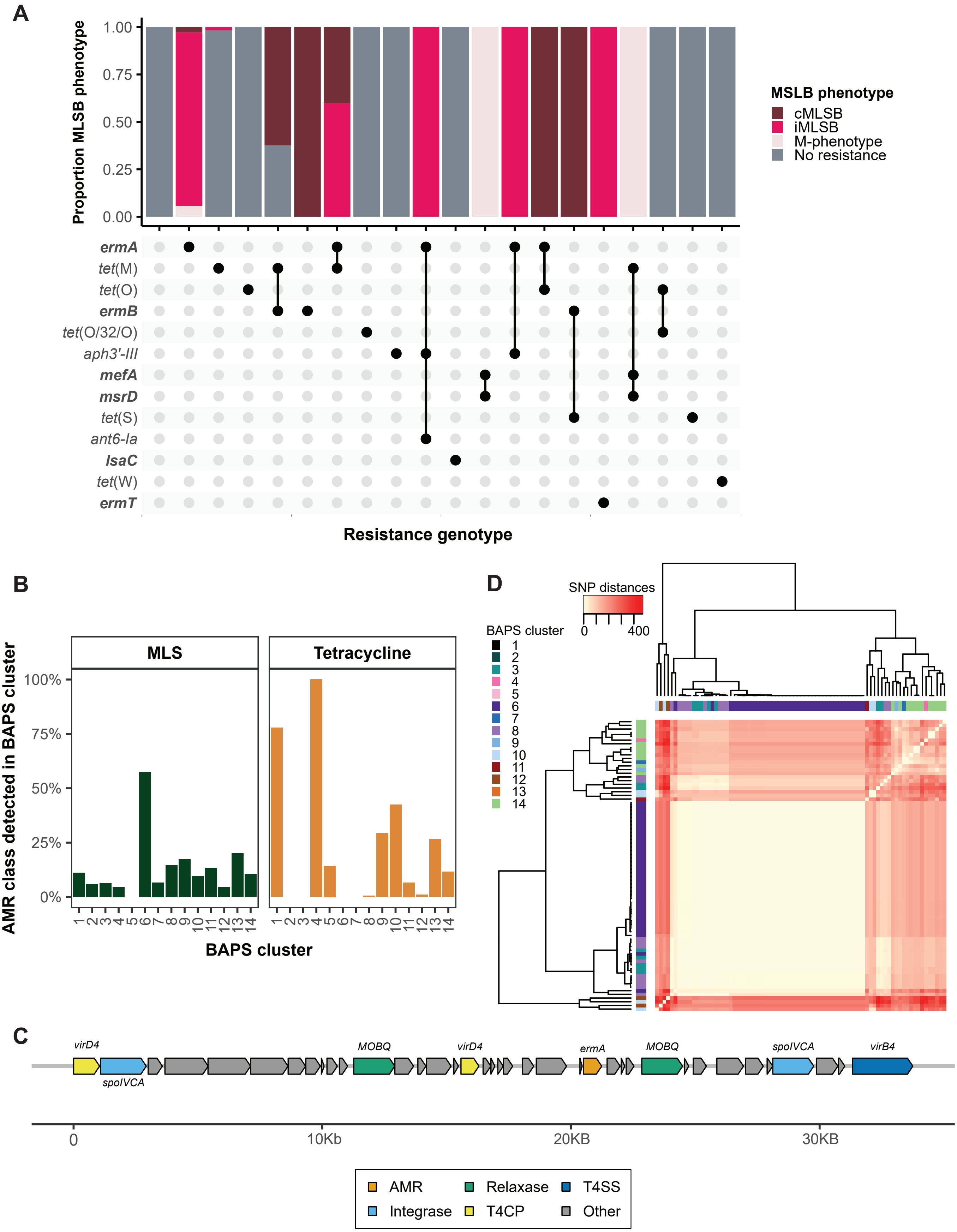


**Supplementary Figure S5. (A)** UpsetR plot visualizing the intersections of resistance gene markers in the iSDSE study population. Resistance genes are indicated in rows and the intersections are indicated in columns. Filled circles indicate presence of the gene. The bar chart above shows the proportion of MLSB (Macrolide-Lincosamide-Streptogramin B) phenotype per resistance genotype. MLSB-resistance relevant genes are highlighted in bold. **(B)** Bar plot visualizing proportion of genomic resistance to antibiotic classes MLS (macrolide, lincosamide, and streptogramin) and tetracycline per BAPS cluster. **(C)** Illustration of the integrative and conjugative element (ICE), ICESDSE1, carrying ermA (~34kb). Colours of the genes indicate antimicrobial resistance genes (AMR) (orange), integrase genes (light blue), relaxases (green), Type IV coupling protein genes (T4CP) (yellow), and Type IV secretions system related genes (T4SS) (dark blue). All other genes are presented in grey. Gene orientation is depicted by the arrow direction. **(D)** Heatmap visualizing pairwise SNP distances between ICESDSE1-positive iSDSE study isolates (n=79). Dendrograms were produced via complete linkage hierarchical clustering. Row and column annotation bars represent BAPS clusters.

## Supplementary Tables

**Supplementary Table S1.** Number of infection episodes per individual for the iSDSE national registry cohort, 1 January 2014 to 31 December 2024.

| Number of infection episodes | Individuals (n = 7,949) | % |
|---|---|---|
| 1 | 7,425 | 93% |
| 2 | 430 | 5.4% |
| 3 | 70 | 0.9% |
| 4 | 16 | 0.2% |
| 5 | 5 | <0.1% |
| 6 | 2 | <0.1% |
| 7 | 1 | <0.1% |

**Supplementary Table S2.** Number of infection episodes per individual for the iSDSE genome sequenced cohort, 17 August 2020 to 18 September 2022.

| Number of infection episodes | Individuals (n = 1,172) | % |
|---|---|---|
| 1 | 1,129 | 96% |
| 2 | 36 | 3.1% |
| 3 | 6 | 0.5% |
| 4 | 1 | <0.1% |

**Supplementary Table S3.** Patient characteristics and genome statistics of SDSE isolates of the genomic study cohort, 17 August 2020 to 18 September 2022.

| **Characteristic** | **n = 1,225** | |
|---|---|---|
| **Sex** | | |
| Female | 457 | 37% |
| Male | 768 | 63% |
| **Age** | | |
| Median (Q1, Q3) | 77 (68, 83) | |
| **Lancefield group** | | |
| A | 3 | 0.2% |
| C | 480 | 39% |
| G | 742 | 61% |
| **Region** | | |
| Denmark | 1,220 | >99% |
| Faroe Islands | 4 | 0.3% |
| Greenland | 1 | <0.1% |
| **Genome size** | | |
| Median (Q1, Q3) | 2,103 kb (2,066 kb, 2,139 kb) | |
| **Average coverage** | | |
| Median (Q1, Q3) | 170 (149, 194) | |
| **Number of contigs** | | |
| Median (Q1, Q3) | 121 (101, 144) | |
| **N50** | | |
| Median (Q1, Q3) | 43 kb (35 kb, 48 kb) | |
| **GC content (%)** | | |
| Median (Q1, Q3) | 39.34 (39.28, 39.43) | |

**Supplementary Table S4**. MLSB-resistance phenotype in the iSDSE isolate study population (n=1,225) with antimicrobial resistance genes detected. MLSB, macrolide, lincosamide, and streptogramin B.

| MSLB phenotype[a] | Isolates | % | AMR genes[b] |
|---|---|---|---|
| iMLSB | 105 | 8.6% | ***ermA***(n=97, 92.4%); ***ermA***/*tet*(M)(n=3, 2.9%); *ant6-Ia*/*aph3'-III*/***ermA***(n=2, 1.9%); *aph3'-III*/***ermA***(n=1, 1.0%); ***ermT***(n=1, 1.0%); *tet*(M)(n=1, 1.0%) |
| cMLSB | 18 | 1.5% | ***ermB***(n=6, 33.3%); ***ermB***/*tet*(M)(n=5, 27.8%); ***ermA***(n=3, 16.7%); ***ermA***/*tet*(M)(n=2, 11.1%); ***ermA***/*tet*(O)(n=1, 5.6%); ***ermB***/*tet*(S)(n=1, 5.6%) |
| M-phenotype | 9 | 0.7% | ***ermA***(n=6, 66.7%); ***mefA***/***msrD***(n=2, 22.2%); ***mefA***/***msrD***/tet(M)(n=1, 11.1%) |
| No resistance | 1093 | 89.2% | No AMR gene(n=993, 90.9%); *tet*(M)(n=56, 5.1%); *tet*(O)(n=28, 2.6%); *tet*(O/32/O)(n=5, 0.5%); *aph3'-III*(n=3, 0.3%); ***ermB***/tet(M)(n=3, 0.3%); ***lsaC***(n=2, 0.2%); *tet*(O)/*tet*(O/32/O)(n=1, 0.1%); *tet*(S)(n=1, 0.1%); *tet*(W)(n=1, 0.1%) |

[a]inducible MLSB-resistance (iMLSB), constitutive MLSB-resistance (cMLSB), macrolide resistance (M-phenotype).
[b]MSLB-relevant AMR genes are highlighted in bold.
